# Network‐level disconnectivity tracks poststroke depressive symptom improvement

**DOI:** 10.1111/pcn.70075

**Published:** 2026-05-14

**Authors:** Aleksi J. Sihvonen, Sonia L. E. Brownsett, David A. Copland, Alistair Walsh, Stephen M. Davis, Geoffrey A. Donnan, Leeanne M. Carey

**Affiliations:** ^1^ Centre of Excellence in Music, Mind, Body and Brain, Cognitive Brain Research Unit (CBRU), University of Helsinki Helsinki Finland; ^2^ Department of Neurology, Neurocenter Helsinki University Central Hospital Helsinki Finland; ^3^ Queensland Aphasia Research Centre, School of Health and Rehabilitation Sciences The University of Queensland Brisbane Australia; ^4^ Surgical Treatment and Rehabilitation Service (STARS) Education and Research Alliance, The University of Queensland and Metro North Health Brisbane Queensland Australia; ^5^ Centre of Research Excellence in Aphasia Recovery and Rehabilitation La Trobe University Melbourne Australia; ^6^ Occupational Therapy, School of Allied Health Human Services and Sport La Trobe University Melbourne Australia; ^7^ Neurorehabilitation and Recovery The Florey Melbourne Australia; ^8^ Melbourne Brain Centre Royal Melbourne Hospital Melbourne Australia; ^9^ Faculty of Medicine Dentistry and Health Sciences The University of Melbourne Melbourne Australia

**Keywords:** connectivity, depression, lesion, lesion network mapping, stroke

## Abstract

**Aims:**

Poststroke depression is the most common psychological condition following a stroke and can severely impact outcome. However, the neurobiological biomarkers associated with recovery of poststroke depression remain unclear. In this longitudinal observational study, we set out to investigate the effect of lesion‐induced focal damage and network‐level disconnectivity, and longitudinal structural connectivity on poststroke depression and its recovery.

**Methods:**

Sixty‐two participants underwent a psychological assessment utilizing the Montgomery–Asberg Depression Rating Scale (MADRS‐SIGMA) at 3 and 12 months post first‐ever stroke. First, we evaluated the relationship between lesions and MADRS scores at the 3 months using voxel‐based lesion‐symptom mapping and lesion network mapping. Next, we assessed the longitudinal relationship between structural connectivity and MADRS scores (change from 3 to 12 months) using multi‐shell diffusion‐weighted MRI data (*n* = 29) collected at both timepoints.

**Results:**

Patient lesions mapped to a connected brain network centered on the left dorsolateral prefrontal cortex (P_FDR_ <0.05). Longitudinal diffusion MRI analysis revealed that increased quantitative anisotropy in the identified structural network was associated with improved depressive symptoms longitudinally (*P* = 0.014), independent of demographic and clinical covariates. Significant associations between the lesions and focal brain structures were not identified.

**Conclusions:**

Depressive symptoms and their improvement mapped to a specific structural brain network. Knowledge of the integrity of this network could prove useful for predicting poststroke depression.

**Clinical Trial Registration:**

START‐PrePARE Australian New Zealand Clinical Trials, www.anzctr.org.au, Registry number: ACTRN12610000987066. EXTEND ClinicalTrial.gov identifier: NCT00887328.

Poststroke depression is the most prevalent psychological condition after stroke, affecting about one‐third of survivors.^1^ Patients with poststroke depression face greater risk of poor recovery, recurrent stroke, reduced quality of life, and higher mortality than those without depression.^2^, ^3^, ^4^ Given the profound individual and societal impact of poststroke depression, accurate identification of vulnerability to depression after stroke is vital. Characterizing the neural underpinnings of poststroke depression is key to understanding it and predicting those at a higher risk; enabling targeted treatment, improved prognosis, better quality of life, and potentially reducing healthcare costs.

Despite many attempts to elucidate the mechanisms of poststroke depression, identifying robust neural predictors has proven challenging. Recent work has argued against a strong role for lesion location: Padmanabhan et al., for example, found that focal lesions alone did not significantly predict depression, whereas lesion locations associated with depressive symptoms were functionally connected to a shared network centered on the left dorsolateral prefrontal cortex.^5^ These findings align with network‐based models proposing that depression arises from dysfunctional processing within specific networks, including the frontostriatal network.^6^, ^7^

However, the literature on focal lesion correlates of poststroke depression is more mixed than often acknowledged. At least three voxel‐based lesion‐symptom mapping (VLSM) studies have reported associations between poststroke depression and focal lesions in regions such as the basal ganglia and dorsolateral prefrontal cortex.^8^, ^9^, ^10^ These studies suggest that discrete lesion locations may contribute to vulnerability to depression, underscoring the need to integrate both focal and network‐level perspectives when interpreting neural predictors of poststroke mood outcomes.

With respect to structural neural networks, diffusion MRI (dMRI) studies have typically examined individual white matter structures^11^ or used graph theory to assess global network topography.^12^ Depression‐related white matter changes have been reported in frontal,^13^, ^14^ temporal,^14^ and callosal^14^ fibers, as well as within the dopaminergic reward circuit.^12^ Yet, most of these findings derive from cross‐sectional analyses, and to our knowledge, no study has examined how longitudinal changes in the structural connectome relate specifically to the recovery of depressive symptoms after stroke. Clearer insight into these structural dynamics would aid clinicians in producing more reliable prognoses and tailoring interventions.

In this study, we combined lesion‐symptom mapping,^15^ lesion network mapping,^16^ and tractography^17^, ^18^ to assess whether focal or network‐level neural architectures underpin poststroke depression and its recovery. Using 3‐month (late subacute) lesion data, we analyzed relationships between focal lesions and their functional network effects on depressive symptoms with VLSM and lesion network mapping (*n* = 62). For a subset with multi‐shell dMRI data at 3 and 12 months poststroke (*n* = 29), we examined structural network changes correlating with the recovery of depressive symptoms longitudinally. Based on prior evidence, including mixed results from lesion studies, we hypothesized that (i) lesions linked to depressive symptoms would not map reliably to specific focal brain areas, but instead (ii) they would map to a common functional network centered on frontal areas, and (iii) structural connectome changes tied to symptom improvement would involve dopaminergic reward pathways projecting to the frontal lobe.

## Materials and Methods

### Participants and study design

This longitudinal cohort study is a part of the ‘STroke imAging pRevention and Treatment (START)’ study approved by Human Research Ethics Committees (HREC 2009.79 and H2010/03588; and for future analysis HREC17Austin281). All participants gave informed written consent in accordance with the Declaration of Helsinki. Inclusion criteria were presenting with acute ischemic stroke, aged ≥18 years. Exclusion criteria were contraindication to imaging with MRI; prestroke modified Rankin Score (mRS) score of ≥2 (indicating previous disability); any terminal illness such that the patient would not be expected to survive more than one year; clinically evident pregnancy; and non‐English speaking.

Sixty‐two participants with a mean lesion volume of 77.8 ml (SD 10.4), who were recruited from five hospital sites in Victoria Australia 2010–2013 (PrePARE substudy^19^) and had advanced neuroimaging data and detailed psychological testing, were included in the current study.

Depressive symptoms were screened using the Montgomery–Asberg Depression Rating Scale Structured Interview Guide (MADRS‐SIGMA)^20^ at two time points, 3 months (mean = 95.8 days, SD = 1.7 days) and 12 months (mean = 369.8 days, SD = 12.4 days) after stroke.

### MRI data acquisition and reconstruction

All neuroimaging data were acquired on a 3T Siemens Trio MRI scanner (Siemens, Erlangen, Germany) with a 32‐channel head coil. MRI data were acquired at 3 months (mean = 95.8 days, SD = 1.8 days), and 12 months (mean = 369.8 days, SD = 12.4 days). For each participant, a high‐resolution structural 3D T1‐weighted image (MP‐RAGE; TR = 1900 ms; TE = 2.55 ms; 256 × 256 matrix, 160 slices, 216 mm FOV; voxel size = 1 mm^3^) and multi‐shell dMRI data with 13 non‐diffusion‐weighted volumes and 85 diffusion‐weighted volumes (25 volumes with *b* = 1000 s/mm^2^ and 60 volumes with *b* = 3000 s/mm^2^) covering the whole brain and brainstem were acquired with following parameters: TR = 8500 ms, TE = 110 ms, voxel size = 2.5 × 2.5 × 2.5 mm^3^. T1‐weighted images were collected for the whole sample (*n* = 62) at 3‐month stage. Multi‐shell dMRI data were collected at 3 months post stroke for a sample of 31 participants, 29 of which also had dMRI data collected at the 12 months follow‐up.

Structural MRI data preprocessing has been described elsewhere.^21^ In short, T1‐weighted images were preprocessed using the Statistical Parametric Mapping software (SPM12, www.fil.ion.ucl.ac.uk/spm/) under MATLAB R2018b, and lesions were manually delineated to the individual T1 images by A.J.S using the MRIcron software package (http://people.cas.sc.edu/rorden/mricron/index.html). Then, T1‐weighted images and lesion images were reoriented, segmented while applying cost function masking, modulated, and normalized to Montreal Neurological Institute (MNI) space, and finally smoothed with an 8 mm full width at the half maximum kernel.

Diffusion data were denoised, corrected for Gibbs ringing artifacts and motion, and reconstructed in the MNI space using q‐space diffeomorphic reconstruction that allows the construction of spin distribution functions in DSI Studio (http://dsi-studio.labsolver.org, version April 7 2021). The restricted diffusion was quantified using restricted diffusion imaging, and quantitative anisotropy (QA) was extracted as the local connectome fingerprint and used in the longitudinal structural analysis. QA is an orientation distribution function (ODF)–based metric that enhances the ability to resolve multiple fiber orientations and improves the accuracy of fiber tracking. Because QA is less susceptible to partial volume effects from free water, crossing fibers, and non‐diffusive tissue components, it provides greater sensitivity than conventional voxel‐ or tract‐based approaches relying on fractional anisotropy (FA).^22^, ^23^

### Voxel‐based lesion‐symptom mapping

VLSM associations were evaluated using nonparametric mapping software (Chris Rorden's NPM, version May 2016) to test whether lesions associated with poststroke depression map to a specific brain region.^15^ To do this, a single whole‐brain analysis was carried out to identify any lesioned brain region associated with higher MADRS scores at the 3‐month stage. All voxels damaged in ≥10% of the participants were included in the statistical analysis.^24^ Correction for multiple comparisons was achieved by using a false discovery rate (FDR)‐corrected P_FDR_ < 0.05 threshold.

### Lesion network mapping

**Fig. 1:**
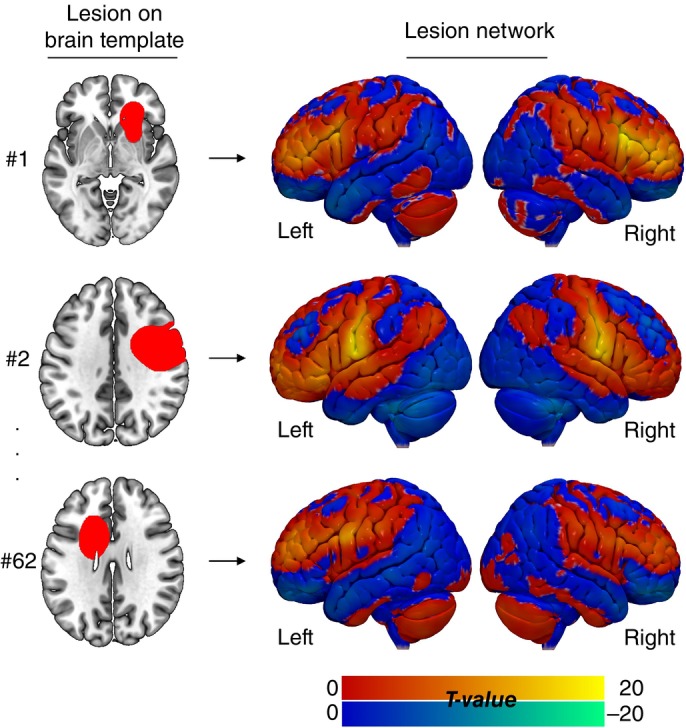
Lesion network mapping approach. Three representative lesions (selected from *n* = 62) traced onto a standard brain atlas. The set of voxels functionally connected to each lesion location is identified (“lesion networks”). Warm colors represent positive functional connectivity with the lesion location; cool colors represent negative functional connectivity with the lesion location.

Lesion network mapping was used as a hypothesis‐driven, region‐of‐interest (ROI) analysis to identify whether lesions associated with depressive symptoms poststroke map to a specific brain network. Functional connectivity between each lesion location mapped to the standard space and all other brain voxels was computed using resting‐state functional connectivity data from 1000 healthy participants (human brain connectome from the Brain Genomics Superstruct Project: https://dataverse.harvard.edu/dataverse/GSP) performed in the Lead‐DBS software package (http://www.lead-dbs.org). This process results in a lesion impacted network for each individual lesion location (Fig. 1). To identify any lesion network voxels within the predefined ROIs that were associated with the depressive symptoms, we performed VLSM^15^ using Nonparametric mapping software (Chris Rorden's NPM, version May 2016), similar to previous lesion network mapping studies.^24^ Based on *a priori* hypothesis and to maximize sensitivity following a published approach,^5^ we limited the lesion network mapping analysis to a bilateral middle frontal gyrus mask based on the automated anatomical labelling atlas 3 (AAL3). Parametric analysis was performed using the 3‐month stage MADRS score. All voxels damaged in ≥10% of the participants were included in the statistical analysis.^24^ Cluster threshold of 100 voxels was used and correction for multiple comparisons was achieved by using an FDR‐corrected P_FDR_ < 0.05 threshold. To assess the robustness of the possible findings to alternative anatomical definitions, lesion network mapping analysis was replicated using Brainnetome atlas^25^ middle frontal gyrus ROIs. In addition, a mask‐free whole‐brain approach was undertaken, allowing us to assess that the observed associations were not driven by a specific parcellation scheme.

### Longitudinal structural network effects

Diffusion MRI‐derived local connectomes were used to identify the longitudinal (3 to 12 months poststroke) structural network effects associated with the lesion network findings identified as underpinning poststroke depressive symptoms using DSI Studio (http://dsi-studio.labsolver.org, version April 7,2021). To do this, the longitudinal structural connectivity from the identified lesion network mapping cluster was calculated using a ROI‐based fiber tracking with 100,000 seeds (step size, random; angular threshold, random; length, 20–300 mm; topology‐informed pruning iterations, 16) using the cluster as a seed. The resulting fiber tracking results were then exported to SPSS (IBM SPSS Statistics 28). To examine associations between longitudinal structural network properties and depressive symptom severity change over time (3 to 12 months), we implemented a linear regression model in which longitudinal QA value was the dependent variable and longitudinal MADRS score change was the predictor of interest. We included the following clinically relevant covariates: MADRS score at 3‐month poststroke stage (to adjust for baseline symptom severity), age, years of education, lesion volume, and MoCA score. These variables were selected because all have established associations with poststroke depression or related affective and cognitive outcomes, and clinical characterizations of depressive symptom profiles in stroke survivors.^26^, ^27^

## Results

### Clinical characteristics of the participants

Across all participants (Table 1), the mean MADRS score was 7.02 at 3 months (SD 7.34) and 5.71 (SD 5.83) at 12 months. Using established MADRS cut‐offs, 28 out of the 62 participants exhibited at least mild symptoms of depression (MADRS score ≥7) at 3 months, with a mean MADRS score of 13.36 (SD 6.47) in this subgroup. Of these 28 participants, 22 showed a longitudinal decrease of at least 2 points from 3 to 12 months—the minimal clinically important difference^28^—with a mean reduction of 12.09 points (SD = 7.19) for this subgroup. At 12 months, 20 participants presented with at least mild symptoms of depression (MADRS score ≥7), with a mean MADRS score of 13.00 (SD = 4.27) in this group. To provide a nuanced and sensitive perspective to the neural network involved in poststroke depressive symptoms, we utilized continuous analyses of MADRS scores rather than binary data, capturing subtle variations and trends.

**Table 1:** Participant characteristics/demographics at both the 3‐month poststroke stage and for those with 12 months follow‐up.

	3 months	12 months
Participants (*n*)	62	29
Age (mean ± SD years)	63.03 ± 12.98	60.64 ± 12.70
Males	18	8
Education (mean ± SD years)	11.62 ± 3.34	12.00 ± 3.31
Days poststroke to MRI (mean ± SD days)	95.46 ± 10.33	369.77 ± 12.36
MADRS scores (mean ± SD)	7.02 ± 7.34	5.71 ± 5.83

*N*, number of participants; SD, standard deviation.

### Lesion‐symptom mapping of depressive symptoms

The results from the voxel‐wise VLSM analyses did not identify any voxels that were significantly associated with poststroke depressive symptoms (*n* = 62).

### Lesion network mapping of depressive symptoms

**Fig. 2:**
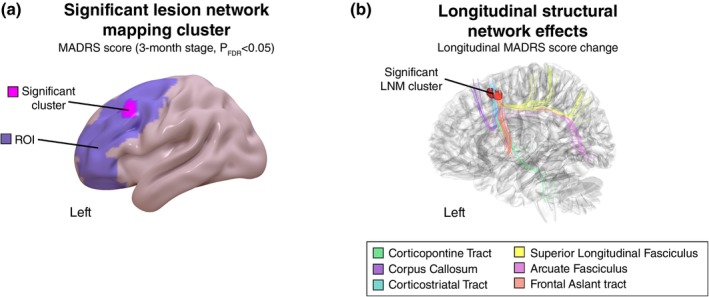
Depressive symptoms network. (a) Parametric lesion network mapping analysis of MADRS scores (*n* = 62), (b) Longitudinal structural network effects of the identified network (*n* = 29). All results are thresholded at P_FDR_ < 0.05. LNM, lesion network mapping; ROI, region of interest.

To identify critical brain areas associated with depressive symptoms poststroke, we conducted lesion network mapping analysis using a normative connectome (Fig. 1) for the MADRS scores at the 3‐month stage (*n* = 62). The ROI‐based analysis revealed that lesions associated with higher MADRS scores (i.e., more depressive symptoms) were significantly connected to a cluster within the left dorsolateral prefrontal cortex (P_FDR_ < 0.05; MNI coordinates: *x* = −38, *y* = 18, *z* = 44, Fig. 2a).

To evaluate whether these findings depended on the ROI definition or the restricted search space, we repeated the analysis using an alternative ROI from the Brainnetome atlas. In addition, we performed a mask‐free whole‐brain approach to permit investigation across the whole brain. Both analyses yielded results consistent with the primary AAL3‐based findings: Brainnetome atlas‐based ROI analysis (P_FDR_ < 0.05; MNI coordinates: *x* = −32, *y* = 18, *z* = 54) and whole‐brain analysis (P_FDR_ < 0.05; MNI coordinates: *x* = −32, *y* = 18, *z* = 54) identified a significant cluster within the left dorsolateral prefrontal cortex significantly connected to lesions associated with higher MADRS scores poststroke. These replication analyses demonstrated comparable lesion network associations with depressive symptom severity (Fig. S1).

### Longitudinal structural network effects

In a multivariable linear model adjusting for baseline MADRS scores, age, years of education, lesion volume, and MoCA scores, longitudinal MADRS change scores remained significantly associated with longitudinal (3 to 12 months) change in QA values within the identified structural network (number of tracts = 122, volume = 5340 mm^3^; *B* = −0.008, SE = 0.003, *t* = −2.68, *P* = 0.014). Greater depressive symptom amelioration over time was therefore related to greater QA increase, independent of demographic and clinical covariates.

In contrast, none of the covariates, age (*P* = 0.735), years of education (*P* = 0.543), lesion volume (*P* = 0.945), or MoCA score (*P* = 0.766), were significantly associated with longitudinal QA change. The baseline MADRS score showed a trend‐level association (*B* = −0.009, SE = 0.004, *t* = −2.04, *P* = 0.054) but did not reach statistical significance.

Taken together, these results indicate that the longitudinal structural connectivity within the examined network, rather than other clinical factors, uniquely predicts reductions in poststroke depressive symptoms.

## Discussion

In this study, we tested the association between poststroke depressive symptoms and depression‐related bilateral frontal network using lesion‐symptom mapping, lesion network mapping, and longitudinal QA‐based tractography analyses. The results suggest that (i) depressive symptoms after stroke did not map robustly to specific focal brain structures, but instead, (ii) were associated with a common functional neural network centered on the left dorsolateral prefrontal cortex, and that (iii) decreases in depressive symptoms over time were associated with increased structural connectivity (i.e., indexed by increased QA in related white matter tracts) within the identified network. These findings support previous lesion‐symptom mapping and lesion network mapping findings on depression^5^ and extend this work by providing convergent longitudinal structural evidence for a network‐level mechanism underlying poststroke depressive symptoms. Importantly, although our lesion network mapping was ROI‐based and hypothesis‐driven, the whole‐brain (mask‐free) analyses replicated the results. Together, these findings offer new evidence that the structural integrity of white matter networks underlying depressive symptoms may capture a continuum of mood dysfunction over time. To our knowledge, this is the first longitudinal study to examine contributions of individual white matter tracts to poststroke depression in combination with lesion‐based metrics.

The network hypothesis of depression proposes that deficits in activity‐dependent neuronal communication underpin the development of depression.^6^ This aligns with the present findings and with recent lesion‐based evidence showing that while lesion location alone may not significantly predict depression, rather, lesions connected to a shared functional network are associated with depressive symptoms.^5^ However, the literature on the contributions of focal lesions remains mixed. Several VLSM studies have reported associations between poststroke depression and focal lesions, but in spatially distributed brain regions such as the basal ganglia and dorsolateral prefrontal cortex bilaterally.^8^, ^9^, ^10^ These findings suggest that in some cohorts, lesion location does contribute to risk for depression, underscoring the need for integrative frameworks that incorporate both focal lesion and network‐level mechanisms. Part of the problem is the lack of appropriate experimental models of depression.^6^ Here, in combination with modern neuroimaging techniques, studying poststroke depression creates a unique opportunity to examine and determine the neural networks that are most critical—and causally linked—to depressive symptoms.^29^, ^30^

Consistent with this perspective, the current study found that stroke lesion locations associated with depressive symptoms were not localized to discrete brain regions but were functionally connected to a shared network centered on the left dorsolateral prefrontal cortex. This supports previous lesion‐mapping evidence implicating the dorsolateral prefrontal cortex as a critical hub in depression.^5^, ^31^, ^32^ Here, individuals with higher MADRS scores (i.e., greater depressive symptoms) demonstrated stronger functional connectivity between their lesion sites and the left dorsolateral prefrontal cortex. The dorsolateral prefrontal cortex plays a crucial role in emotion regulation^33^ and executive functions such as working memory, attention, decision‐making, and cognitive flexibility,^34^ domains frequently disrupted in major depressive disorder. Moreover, stroke survivors with depression have been shown to exhibit dysfunction of the dorsolateral prefrontal cortex^34^ and its altered connectivity between other brain regions involved in emotional processing, such as the limbic system.^35^ From an altered connectivity perspective, associations between basal ganglia lesions,^9^ especially the globus pallidum,^10^ and depressive symptoms may arise because such lesions disrupt prefrontal network connectivity,^36^ rather than because they directly damage the dorsolateral prefrontal cortex^10^ itself.

The current study also provided novel longitudinal structural evidence for a neural network associated with reductions in depressive symptoms poststroke. Improvements in mood were associated with increases in white matter integrity within a neural network comprising frontal association pathways, commissural pathways, and projection pathways linking frontal cortices with the striatum. This aligns with a previous meta‐analysis of resting‐state functional connectivity studies of major depressive disorder revealing reduced connectivity within multiple functional networks.^37^ Specifically, we found that a longitudinal decrease in depressive symptoms was associated with increases in QA in the corpus callosum, left frontal aslant tract, superior longitudinal fasciculus, arcuate fasciculus, corticostriatal tract, and corticopontine tract. Previous studies utilizing structural and functional connectivity data have associated alterations in the corpus callosum,^38^ especially its frontal fibers running through the genu^39^ similar to the current results, superior longitudinal fasciculus,^40^ arcuate fasciculus,^41^ corticostriatal tract,^42^ and corticopontine tract^43^ with depression and major depressive disorder.

By adopting a multimodal MRI approach including longitudinal structural connectivity, this is the first study to identify a potential role for the frontal aslant tract in poststroke depression. This tract interconnects the dorsolateral prefrontal cortex, anterior cingulate, and anterior insula.^44^ While its precise function is still being established, it is known to contribute to motor planning, speech, and language.^45^ Emerging evidence links abnormalities in this tract to executive dysfunction,^45^ a domain closely tied to depression.^46^ However, the specific involvement of the frontal aslant tract in depression is not yet fully understood. Given that the frontal aslant tract links multiple regions implicated in mood regulation,^37^ damage or subsequent reorganization of this tract may plausibly contribute to poststroke depressive symptoms, as suggested by our findings.

This study has several limitations. First, it must be acknowledged that as our sample size is relatively modest, particularly in the longitudinal dataset, future larger‐scale longitudinal studies are needed to assess the out‐of‐sample validity of the current results. Second, lesion network mapping uses functional normative data that assume the patterns of connectivity in healthy individuals are similar to a stroke survivor prior to their brain insult. This assumption appears reasonable, however, given the success and robustness of lesion network mapping across various symptoms.^5^, ^16^, ^24^ Third, the current study focused on lesion connectivity and the structural connectome to explain poststroke depression. We cannot therefore determine if the results reflect causality or a compensatory adaptation, or indeed a combination of the two. A further limitation lies in our use of frontal ROIs for the primary lesion network mapping analysis. Although hypothesis‐driven, such ROI selection may bias the analysis toward detecting connectivity patterns centered on the prefrontal network and could overlook relevant non‐prefrontal networks. Notably, the unbiased, whole‐brain mask‐free mapping reproduced the same core network cluster, indicating that the observed dorsolateral prefrontal cortex‐anchored network did not depend on ROI selection and is most likely a genuine feature of the depression‐related network.

## Conclusion

We provide evidence that, rather than focal neural structures, poststroke depressive symptoms are associated with disruption to a neural network involving multiple white matter pathways, centering on the left dorsolateral prefrontal cortex. By using multimodal MRI data as well as longitudinal dMRI data, we provide novel structural evidence supporting the relevance of this network in the recovery trajectory of poststroke depression. These results aid our understanding of the network hypothesis of depression, and the structures involved in the recovery of depressive symptoms. This information may, in the future, help clinicians to provide more reliable prognoses of poststroke depression and recovery and inform the development of targeted treatments for this common and debilitating consequence of stroke.

## Disclosure statement

The authors have no conflicts of interest to declare.

## Author contributions

A.S. contributed to conceptualization, data curation, formal analysis, funding acquisition, methodology, visualization, writing – original draft preparation, writing – reviewing and editing. S.B. contributed to conceptualization, visualization, writing – original draft preparation, writing – reviewing and editing. D.C. contributed to conceptualization, funding acquisition, resources, supervision, writing – original draft preparation, writing – reviewing and editing. A.W. contributed to conceptualization, resources, writing – original draft preparation, writing – reviewing and editing. S.D. contributed to conceptualization, writing – original draft preparation, writing – reviewing and editing. G.D. contributed to conceptualization, writing – original draft preparation, writing – reviewing and editing. L.C. contributed to conceptualization, funding acquisition, investigation, methodology, project administration, resources, supervision, writing – original draft preparation, writing – reviewing and editing.

## Funding

We acknowledge financial support for the conduct of the research from the Commonwealth Scientific Industrial Research Organization (CSIRO) of Australia Flagship Collaboration Fund through the Preventative Health Flagship scheme and support for the analysis, writing, and researchers from the National Health and Medical Research Council (NHMRC; #1104194; #2004443; #1113352), NHMRC‐funded Centres of Research Excellence in Aphasia Recovery and Rehabilitation (#1153236) and Stroke Rehabilitation and Recovery (#1077898), Finnish Cultural Foundation (#191230), Maire Taponen Foundation, Orion Research Foundation sr, Research Council of Finland (#368008), and Signe and Ane Gyllenberg Foundation. The funding sources had no role in the conduct of this study or the writing of this report.

## Supporting information


**Figure S1** Network associated with depressive symptoms. Parametric lesion network mapping of MADRS scores (*n* = 62) performed using AAL3 and Brainnetome middle frontal gyrus ROIs alongside a mask‐free whole‐brain approach. Statistical maps are thresholded at P_FDR_ <0.05.

## Data Availability

Research data are not shared.
