# Supplementary material for: Network‐level disconnectivity tracks poststroke depressive symptom improvement
Source: Psychiatry Clin Neurosci. 2026 May 14;80(8):676–81. doi: 10.1111/pcn.70075 (PMC13447115; doi:10.1111/pcn.70075)
Supplement: Supplementary file 1 [file PCN-80-676-s001.docx]

# SUPPLEMENTARY MATERIAL

# Network-level disconnectivity tracks poststroke depressive symptom improvement

Aleksi J. Sihvonen^1,2,3,4^ (aleksi.sihvonen@helsinki.fi)
Sonia L.E. Brownsett^3,4,5^ (s.brownsett@uq.edu.au)
David A. Copland^3,4,5^ (d.copland@uq.edu.au)
Alistair Walsh^6,7^ (divide5@gmail.com)
Stephen M. Davis^8,9^ (stephen.davis@mh.org.au)
Geoffrey A. Donnan^8,9^ (geoffrey.donnan@unimelb.edu.au)
Leeanne M. Carey^5,6,7^ (l.carey@latrobe.edu.au)

1 Centre of Excellence in Music, Mind, Body and Brain, Cognitive Brain Research Unit (CBRU), University of Helsinki, Finland
2 Department of Neurology, Neurocenter, Helsinki University Central Hospital, Finland
3 Queensland Aphasia Research Centre, School of Health and Rehabilitation Sciences, The University of Queensland, Brisbane, Australia
4 Surgical Treatment and Rehabilitation Service (STARS) Education and Research Alliance, The University of Queensland and Metro North Health, Queensland, Australia
5 Centre of Research Excellence in Aphasia Recovery and Rehabilitation, La Trobe University, Australia
6 Occupational Therapy, School of Allied Health Human Services and Sport, La Trobe University, Australia
7 Neurorehabilitation and Recovery, The Florey, Australia
8 Melbourne Brain Centre, Royal Melbourne Hospital, Melbourne, Australia
9 Faculty of Medicine Dentistry and Health Sciences, The University of Melbourne, Australia

**Corresponding author:** aleksi.sihvonen@helsinki.fi

**Supplementary Figure 1.** Network associated with depressive symptoms. Parametric lesion network mapping of MADRS scores (n = 62) performed using AAL3 and Brainnetome middle frontal gyrus ROIs alongside a mask‑free whole‑brain approach. Statistical maps are thresholded at P_FDR_ < 0.05.
